# Enhancing patient and public contribution in health outcome selection during clinical guideline development: an ethnographic study

**DOI:** 10.1186/s12913-022-07736-6

**Published:** 2022-03-18

**Authors:** Alice M. Biggane, Bridget Young, Paula R. Williamson, Erin Whittingham, Jessie Cooper

**Affiliations:** 1 Roche Products Ltd, Welwyn Garden City, UK; 2 Department of Public Health, Policy and Systems, Institute of Population Health, University of Liverpool, Liverpool, UK; 3 Department of Health Data Science, Institute of Population Health, University of Liverpool, Liverpool, UK; 4 Liverpool Health Partners, Liverpool, UK; 5 Public Involvement Programme, National Institute of Health and Care Excellence (NICE), Manchester, UK; 6 Division of Health Services Research and Management, School of Health Sciences, City, University of London, London, UK

**Keywords:** Ethnography, Patient and public involvement, Health outcomes, Clinical guidelines, Guideline development, Lived experience

## Abstract

**Background:**

Patient and public involvement (PPI) is a cornerstone in enhancing healthcare research and delivery, including clinical guideline development. Health outcomes concern changes in the health status of an individual or population that are attributable to an intervention. Discussion of relevant health outcomes impacts the resulting clinical guidelines for practice. This study explores how the input of PPI contributors at the National Institute of Health and Care Excellence (NICE) is integrated into guideline development, particularly in relation to health outcome selection.

**Methods:**

The study used an ethnographic methodological approach. Data comprised: observations of committee meetings, scoping workshops and training sessions, and in-depth interviews with PPI contributors, health professionals and chairs from clinical guideline development committees. Data were analysed thematically.

**Results:**

PPI contributors’ input in the guideline development process was often of limited scope, particularly in selecting health outcomes. Key constraints on their input included: the technical content and language of guidelines, assumed differences in the health-related priorities between PPI contributors and health professionals, and the linear timeline of the guideline development process. However, PPI contributors can influence clinical guideline development including the selection of relevant health outcomes. This was achieved through several factors and highlights the important role of the committee chair, the importance of training and support for all committee members, the use of plain language and the opportunity for all committee members to engage.

**Conclusions:**

Lay member input during the outcome selection phase of clinical guideline development is achievable, but there are challenges to overcome. Study findings identify ways that future guideline developers can support meaningful lay involvement in guideline development and health outcome selection.

**Supplementary Information:**

The online version contains supplementary material available at 10.1186/s12913-022-07736-6.

## Introduction

Clinical guidelines are “systematically developed statements to assist practitioner and patient decisions about appropriate health care for specific clinical circumstances” [1]. If successfully designed and implemented, clinical guidelines should standardise practice by reducing variation in care across health settings [2, 3]. However, poorly developed guidelines can compromise the quality of care and result in suboptimal, ineffective or even harmful practices [4]. Clinical guideline development follows rigorous methodology which often includes systematic reviews of research evidence. To ensure that the evidence is translated into meaningful guidelines, it is essential that relevant evidence is sought and considered in the context of the everyday realities of healthcare service, use and delivery [5, 6]. The involvement of key stakeholders, including patients and the public, in the guideline development process is important to ensure that guidelines are applicable to all those who will use or be affected by them [7–9].

Patient and public involvement (PPI) is defined as clinical research and development “being carried out ‘with’ or ‘by’ members of the public” not just “‘to’, ‘about’ or ‘for’ them” [10]. PPI contributors, often also known as patient research partners, are often seen as members of the research study team and actively contribute to the design, conduct and dissemination of a health research study (43). PPI, is widely seen as central to enhancing the value and impact of healthcare research and delivery [11], and this includes clinical guideline development [7]. PPI is recommended or required by numerous global bodies and organisations [12–15] such as the National Institute of Health and Care Excellence (NICE), who develop clinical guidelines for health and care for use by the National Health Service (NHS) in England [15]. NICE term and describe PPI in their processes as “lay involvement”. PPI is an evolving area, and there is still debate about its definition, methods, operations, integrity and ethical standards [16]. As a result, PPI can take a range of different forms, which is informed by multiple layers, including whether the PPI is occurring in the research or clinical development setting. Therefore, a key consideration concerning PPI, is ensuring that the process can adapt to the specific context and setting, and that it works for the people and groups concerned. Previous research describes a need to ensure that patients are equal stakeholders in an expert-dominated environment, and that their lived experience and knowledge is integrated into the research and development process [17–19]. Failure to engage meaningfully with patients and members of the public can lead to tokenism, described as the “superficial and disingenuous” inclusion of small numbers of patients, with limited involvement and impact on the process [20–23].

Tokenistic PPI has the potential to limit the influence of involving patient and members of the public equally and meaningfully in the design and delivery of quality healthcare, including, but not limited to,, the development of clinical guidelines. Further, tokenistic PPI can also have damaging effects on the stakeholders and processes involved, which in turn negatively impacts healthcare and ultimately, societal gain. There have been calls to move beyond the *“narrow and exclusive approach”* often associated with tokenistic PPI and to have a *“critical appraisal of evidence and a debate about the focus and methods of involvement”* to improve it [24]. Organisations, like NICE, have responded to the need for meaningful patient and public input in guideline production by developing various practices and mechanisms to enable and support PPI in their processes [25].

In recent years, social scientists have examined the production of clinical guidelines [26], and how absences of evidence in key areas of the guidance are managed [27]. This paper draws on data from a wider ethnographic study which aimed to examine how PPI contributors are involved in and experience NICE guideline development, and crucially, to understand how their inputs affect the guideline development process. Specifically, this wider study aimed to characterise the processes involved in the integration and contribution of lay members’ views within clinical guideline selection and to examine how lay members negotiate and influence the outcomes chosen in clinical guideline selection.

This paper examines the process surrounding how decisions are made about which health outcomes to use to inform and develop clinical guidelines, focusing on the lay member role. Health outcomes concern changes in the health status of an individual or population that are attributable to an intervention [28]. This phase warrants attention as the outcomes selected will determine what evidence informs the guideline development process, and thus shape the final recommendations. Previous research suggests health professionals have overlooked, or deemed insignificant, health outcomes that were later identified as important to patients [29, 30], pointing to the need for meaningful PPI in this phase of guideline development.

## Methods

### Research design

This paper is based on data from an ethnographic study called the ‘The INVoLVED Study’—Investigating Lay-members’ Views in Clinical Guideline Development. The study used observations and qualitative interviews to examine key stakeholders’ activities and experiences during NICE clinical guideline development [31]. An ethnographic methodology is particularly suited to understanding what shapes lay involvement and the process of clinical guideline development, as it enables the researcher to become immersed in the activities of groups or organisations in their natural setting, in this instance, in committee meetings at NICE [32]. NICE was an appropriate setting for this research as it plays a vital role in the development of evidence based clinical guidelines in England via expert committees that review evidence. These committees comprise health professionals, care providers and “lay members” [33]. Lay members are individuals with personal experience of using health or care services, or from a community affected by the guideline. Including “lay members” is central to how NICE facilitate PPI within clinical guideline development.

In what follows, including the Results and Discussion sections, we use the term ‘lay member’ as defined by NICE to refer to patients, carers, service users and people from organisations who represent these groups. We use ‘health professional’, as defined by NICE to refer to clinicians and clinical academics.

The study was reviewed and approved by the University of Liverpool ethics committee in October 2017 (reference: 2025).

### Data collection and analysis

The lead researcher (AMB) conducted in-situ observations of clinical guideline meetings, scoping workshops, and lay member training sessions over a 12-month period in 2017/18 (Table 1). In total, observations comprised 22 meetings tallying over 230 h of observational fieldwork (see Table 1). Most observations were of guideline meetings (*n* = 18), while others were scoping (*n* = 3) and training workshops (*n* = 1). AMB observed meetings in relation to five different guidelines over this period (guidelines observed included GC1, GC4, GC5, GC6 and GC9, see Table 1). However, there was a specific focus on two of these guidelines, GC4 and GC9. These guidelines were selected as their development timelines allowed AMB to regularly observe meetings (GC4 from the first to the final meeting; GC9 from the first to a near final meeting) and they were in two differing health areas (GC4 was cancer and GC9 was obstetrics). NICE placed no limits or restrictions on what meetings AMB could attend and observe, similarly, AMB had access to all the same documentation as the committee members. Thus, in the initial stages of this study, AMB observed and documented as much as possible, in keeping with the ethnographic nature of the study. However, as AMB and the research team became more familiar with the data and the study, the observations became more refined and focused specifically on those processes and interactions which influenced lay member input and interactions, in keeping with wider aims of the study from which this current paper is informed. During the observations AMB also conducted ethnographic interviews (spontaneous, informal conversations) with various committee and technical team members. Committee members gave written consent for the first observations and verbal consent at subsequent meetings. Observations and ethnographic interviews were first recorded in handwritten pseudo-anonymised fieldnotes, which were then written up electronically. AMB also collected relevant documents such as agendas, meeting minutes, and reports and used these as a memory aid when writing up the fieldnotes. It was these resulting fieldnotes which formed the basis for subsequent analysis and interpretations.

**Table 1:** The setting and focus of the in-situ observations, including the number of observations made in each setting. A total of five different guidelines were observed, GC1, GC4, GC5, GC6 and GC9. ^a^Several meetings occurred over 2 days

Number of observations	Guideline Topic	Focus of observations	Breakdown of observations
***In- depth guideline committee (GC) observations (n* = *15)***			***All full day meetings*** ***9.30am- 5 pm***
8	Cancer (GC4)	The inclusion of lay members in developing the guideline and the interactions and processes surrounding their inclusion	8 out of 8 possible meetings 12 full days^a^
7	Obstetrics (GC9)		7 out of 13 possible meetings 8 full days^a^
***Additional guideline committee (GC) meetings (n* = *3)***			***All full day meetings*** ***9.30am-5 pm***
1	Cancer (GC6)	The inclusion and interactions of lay members within the specific meeting	1
1	Cardiovascular health (GC5)		1
1	Gynecology (GC1)		1
***Scoping workshops*** ***(n* = *3)***			***All half-day meeting*** ***3 h meetings***
1	Dermatology	The inclusion of lay members in scoping of the guideline. The interactions and processes that occurred	1
1	Mental health		1
1	Rehabilitation		1
***Training workshops*** ***(n* = *1)***			***All full day meetings*** ***9.30am-5 pm***
1	Lay member training session	Training and advice NICE provided to lay members and their interactions on the day	1
**Total meetings: *n* = 22**			**Total hours: 230 approximately**

In addition to observations and ethnographic interviews, AMB conducted eighteen in-depth, semi-structured interviews with lay members, health professionals and committee chairs involved in the guideline development (Table 2). Interviewees were lay members (*n* = 14) from nine different guideline committees, health professionals (*n* = 2) from two guideline committees and committee chairs (*n* = 2) from two guideline committees (see Table 2). These interviews explored participants’ understanding and experience of the guideline development process, and the role and influence of the lay members (Additional file 1). Interviews were conducted face to face (*n* = 11) or via telephone (*n* = 7), and all interviewees gave either written or verbal informed consent before beginning the interview. Interviews, which lasted 75 min on average, were audio-recorded, transcribed verbatim, and pseudo-anonymised before being analysed.

**Table 2:** In-depth, semi-structured interviewee demographic characteristics

	***Pseudonym***	***Gender***	***Committee***	***Committee Role***
1	Joan	Female	GC1	Lay member (patient)
2	Antonia	Female	GC1	Lay member (patient)
3	Grace	Female	GC2	Lay member (carer and associated with relevant patient charity)
4	Frances	Female	GC3	Lay member (senior employee of relevant national patient charity)
5	William	Male	GC4	Committee chair
6	Richard	Male	GC4	Lay member (patient)
7	Lisa	Female	GC4	Lay member (senior employee of relevant national patient charity)
8	Henry	Male	GC4	Health professional
9	Greg	Male	GC5	Lay member (patient)
10	Julian	Male	GC5	Lay member (patient)
11	Ben	Male	GC6	Lay member (patient)
12	Dylan	Male	GC6	Lay member (carer)
13	Ann	Female	GC7	Lay member (patient)
14	Mary	Female	GC8	Lay member (patient)
15	Cecilia	Female	GC9	Health professional
16	Andrew	Male	GC9	Committee chair
17	Ruth	Female	GC9	Lay member (carer)
18	Jennifer	Female	GC9	Lay member (carer)

Lay committee members that were sampled varied in age (from approximately mid 20 s – late 60 s), lived experience (as a patient, a carer or relevant charity representative (see Table 2.), NICE experience (first-time committee member vs. previous NICE committee member) and occupations (ranging from currently employed to retired, across a range of professional and manual sectors).

Health professional committee members that were sampled ranged in age (approximately 30–65 years), NICE committee experience (first-time committee member vs. previous NICE committee member) and covered a range of health professional roles (doctors, surgeons, nurses, dieticians, etc.).

Reflecting with our wish to provide a practical, actionable account and recommendations, we took a pragmatic theoretical standpoint to consider interpretive-qualitative knowledge [34] we collected throughout the study. This approach allows research teams to be flexible and open to a blend of epistemologies and procedures as appropriate for their study. Thus, we were also eclectic, and drew upon the interpretivist paradigm, recognising multiple realities and interpretations [35, 36], and that the researcher and their study population are interdependent and mutually interactive [37].

Procedurally, data analysis was iterative and thematic, following Braun and Clarke’s framework [38]; it focused on identifying how lay member input in guideline development committees was structured and patterned. This involved considering the data from the study, both within the context of the particular guideline and setting in which data were collected and also within NICE’s framework and processes to implement lay member input. Further, we explored the influences and challenges around lay member input as we observed them and sought to put this into the wider context of NICE practices and the available literature on PPI in health care settings. AMB led the analysis, first reading and annotating the observation and ethnographic interview fieldnotes and interview transcripts, then coding the data and grouping similar codes together to identify recurring patterns, and organise these into themes and categories [38, 39]. The use of multiple methods and data sources, enabled triangulation and thus, helped us in developing a comprehensive understanding of the processes [40]. JC, EW and BY reviewed transcripts and fieldnotes and regularly discussed the themes and categories with AMB to refine the analysis. The study team agreed that data saturation had been reached after 230 h of observation (including ethnographic interviews) and eighteen semi-structured interviews. Microsoft Word was used to facilitate the organization and processing of the coding and analysis [41].

## Results

As previously stated, our focus was mainly on the health outcome selection phase of guideline development and lay member involvement in this selection. We show that while most lay members became involved in other aspects of the clinical guideline development, their involvement in selecting health outcomes was relatively limited. How the role of lay members was perceived, the timeline of the guideline development process, and the medical and scientific technicality of the guideline content all had a part in constraining lay member involvement. Nevertheless, we did observe four instances of lay members having direct or indirect influence on health outcome selection. In presenting the findings we draw on these four instances to suggest ways that lay member involvement in outcome selection could be facilitated. At various points of the guideline development process, we also found that the involvement of health professionals was limited in similar ways to that of lay members. Thus, in some instances, we report findings in relation to committee members generally, rather than lay members exclusively.

### Outcome selection and lay member involvement

Procedurally, lay member input in health outcome selection during NICE clinical guideline development can occur at two junctures: i) scoping workshops, where the remit and scope of the guideline is discussed and agreed upon, and ii) committee meetings, where the guideline is developed in line with the output of the scoping workshops, by reviewing the relevant evidence. During the early committee meetings, the technical team, which comprised systematic reviewers and technical analysts employed by NICE, devise evidence review protocols in consultation with the committee members. To populate these protocols with appropriate search terms, the technical team follow the PICO (patient/problem/population, intervention, comparator and outcome) framework. Thus, it is during these early meetings that health outcomes are selected. The resulting literature and evidence are then discussed and contextualised at subsequent meetings. In theory, all members of the committee, including the lay members, can be involved in each step of the guideline development process.

NICE Public Involvement Programme (PIP) facilitate training sessions, to introduce lay members to the guideline development process, including topics such as understanding scientific evidence and how it is used, what are outcomes, and what is the role of lay members in the guideline development process. During our observation of one of these training sessions, lay members were also advised of various resources and support available regarding guideline development and were provided with examples illustrating the impact of lay members in previous guideline developments. When interviewed, lay members largely described this training as *“helpful”* and *“empowering”*, praising the way their role, the process and the scientific terminology were explained.

During the observed training session, the PIP team spoke about the importance of lay member input in health outcome selection:

*There was some time dedicated to explaining “outcomes” and how the “lay member voice and input is needed” in deciding what outcomes to search for in various literature sources. A slide on the PowerPoint presentation read: “Protocol stage is a good opportunity for lay members to identify outcomes of the treatment, activity or care that are important to people using services or carers.” Further to that lay members were advised to “be specific, evidence reviews are resource intense”, and that “usually there are 3-4 main outcome measures.” PIP also provided some examples explaining what outcomes are. Lay members appeared to be actively taking notes directly onto their handouts during this session. (*Fieldnotes from the lay member training session.)

However, despite this emphasis by the PIP team and lay members’ accounts that these sessions were generally useful, lay members largely did not recall the focus on the importance of their role in health outcome selection. When asked, no lay members interviewed said the training session had influenced their involvement in health outcome selection, and indeed, most did not mention health outcomes as an area they were, or even wanted to be involved with. Moreover, during observed committee meetings, lay members rarely participated in discussions about health outcomes or their selection. When asked who he thought was most involved in setting the evidence review protocols (which is where outcome selection occurs through the PICO format as outlined previously), Andrew, the chair from GC9, said:

“*I think probably the NICE technical team, followed by the health professionals with specific expertise on the committee would probably be the ones who had the most influence on PICO, […] the lay members get involved in the later discussion.”* Andrew, GC9, (interview)

Echoing Andrew’s description, observations of GC4 and GC9 meetings indicated that the technical team led most of the early phase meetings by introducing prepared drafts of evidence review protocols for each review question and asking the committee to comment. Generally, committee members, both health professionals and lay members, only became involved when their specific expertise was relevant to the review question. Frances, from GC3, was the only lay member who recalled learning about evidence review protocols and health outcome selection at the lay member training session. She characterised the protocol setting and thus, health outcome selection as something that was outside her remit, as a: *“system the technical team would go through”.* According to Frances, both lay members and health professionals did not become involved until the later phase when they started to look “*at the* (resulting) *evidence statements and use those to decide what the overall recommendation was.”* Observations of committee meetings and subsequent interviews largely indicated that the input of health professionals and lay members varied at different stages of guideline development, and that the input of both groups was limited at the health outcome selection stage.

#### Lay members’ views of their role

While lay members mostly saw health outcome selection as the preserve of the technical team, they did describe having an indirect role in the clinical guideline development process. This role involved them presenting the perspectives of patients whose care and treatment would be influenced by the clinical guideline. For example, Richard a lay member from GC4 spoke on multiple occasions about his *“motivation”* for joining the committee, commenting that:

*“The whole* (patient) *journey through the cancer thing […] I thought there was a colossal void with a lot of very excellent stage posts during the process, during the treatments, during the investigations and so on, but there were big gaps in-between and it is a pretty desolate landscape when you are on the other* (patient) *side.”* Richard, GC4, (interview)

Richard’s hope in joining the committee was to explain this *“void”* from a patient perspective, thereby helping to ensure that the guideline would improve the *“journey”* for future patients. Other lay members made similar comments, describing an overarching aim to humanise the guideline by emphasising the patient experience.

While lay members did not, therefore, directly comment on or suggest health outcomes, by providing their patient experience they were able to indirectly contribute to health outcome selection. For example, Joan, a lay member from GC1, described how her experiential knowledge offered a different perspective on an intervention to that of health professionals:

*“The clinician said oh it is just a simple test [...] And I said excuse me it’s not a simple test and so* (technical team member) *said ok explain to us and so I gave the graphic detail of what it’s really like, and the clinician was saying yes actually that is true. So, I thought oh gosh I have got something to add here. […] So, for the researchers to understand precisely perhaps even visualise I think that was helpful for them trying to weed out quite what the key search terms should be to get a bigger understanding of what we’re trying to say.”* Joan GC1, (interview)

Due to her involvement early in the process, and by sharing her lived experience, Joan was able to influence the committee and subsequently ensure lay member input in the search terms used, including outcomes. However, Joan was the only lay member interviewed to discuss her active role and participation in this early phase of clinical guideline development.

### Understanding the challenges surrounding lay involvement

As described above most lay members did not see health outcomes as part of their role and most did not directly participate in the selection of outcomes during clinical guideline development. Below, we draw on the data to illustrate three reasons why lay member involvement in health outcome selection was limited.

#### Guideline development timeframe

The data indicated that lay members and health professionals needed time to familiarise themselves with the guideline development process and with each other. As we show below, the lack of such time restricted the input of lay members in health outcome selection. The clinical guideline development follows a largely linear process, with little time for revisiting tasks and items if questions arise at later points when members are more familiar with the process and one another.

During the early phase meetings, where lay members learned about the process and became familiar with other members of the committee, they were often silent and did not get involved in setting evidence review protocols. Various committee members spoke of the time it took them to ‘find *their feet”* in terms of understanding and being able to participate in the guideline development process. Grace, a lay member from GC2 further elaborated: *“I felt a bit lost in it all and it took me probably a good 9 months before I really understood how the process worked”*. Similarly, health professionals described the early phase meetings as *“a learning process”* while Cecilia, a health professional made a similar point:

*“Some things probably could have beared repeating. […] a lot of the time, silence is taken as an indicator that yes you are fine with everything but it can often mean I am not really sure what is going on but I am just going to just keep on listening, see if I can pick it up.”* Cecilia, GC9 (interview).

Committee members became more involved in proceedings as they became familiar with the process and its content, as well as becoming comfortable with each other. As the following fieldwork excerpt indicates, discussions observed in later phase GC4 and GC9 meetings stood in contrast to those in the earlier phases of guideline development:

*A number of health professionals became quite animated and involved in discussion, they had been largely silent in the meetings up to this point. A few dynamics have changed i) we are now into the evidence discussion phase so they can offer their interpretations, ii) committee members are visibly more comfortable with the process and with each other; they are now chatting together more regularly during meetings and break time. During discussions they appear to engage more with each other, challenging and supporting what has been said.* Fieldnotes from GC4, meeting 5

All committee members therefore became more engaged and vocal as the process unfolded. However, this often meant that any need for different avenues of scientific inquiry or search terms for evidence review protocols only became apparent in later meetings. This became an issue when, during subsequent evidence discussions and recommendation writing meetings, committees requested evidence which differed from what had been originally agreed in the earlier evidence review protocols. Joan a lay member from GC1 was one of the committee members to describe her *“difficult”* experience of this:

*“We have asked for another review that has been rejected and I find that NICE can be quite inflexible and […] saying well those were your search terms* (including outcomes)*, so that is the end of it.”* Joan, GC1 (interview)

According to Joan, the technical team sometimes responded inflexibly to requests for further evidence searches. Our interpretation from meeting observations was that the technical team were unable to respond to such requests due to limitations on time and personnel. In GC9 and GC4 we observed similar tensions, with committee members requesting further evidence, with the technical team unable to act on these requests. In these instances, the committee were usually tied to the original search terms and protocols agreed during the early phase meetings. In turn, lay members were limited in their later involvement, if the resulting evidence was not relevant and clear to them.

#### Technical content of guidelines

A further issue in lay members’ involvement related to the highly technical nature of the language used by the committees and in the literature provided. For example, one of the guideline development review questions asked "* ‘what is the optimal dose and fractionation schedule for people with localised* (type removed) *cancer* (cancer grading and staging removed) *who are treated with radical radiotherapy?’* Lay members unsurprisingly felt the technical nature of discussions inhibited them from participating. This technical content comprised terminologies, abbreviations and topic content which lay members believed were largely only accessible to health professionals or those with specialised knowledge.

When interviewed lay members often made statements such as, *“half the time I have no idea what they are talking about”* (Joan, lay member, GC1) while some expressed feelings of “*frustration”* or commented that their involvement amounted to “*tokenism”* (Richard, lay member, GC4). Observations of committee meetings further point to this*:*

*“Ruth, a lay member, was invited to speak and I was struck by the change in her body language from earlier in the day, she looked annoyed, no longer smiling or trying to engage with the other committee members. She talked about the difficulties the lay members had that morning and in previous meetings in understanding the review processes and related terminology and discussion content. She said that their lack of understanding means “they can’t contribute as might be expected”. Rebecca, a health professional, replied that “it’s very difficult” to talk about the content in other terms as they are the topics that they have to review, to which the other health professionals nodded in agreement.”* Fieldnotes from GC9, meeting 4.

Ruth noted here the recurring difficulties lay members had in understanding the clinical guideline topic and content, which limited their overall involvement. In response to such difficulties, Jennifer another lay member from GC9 described negotiating an alternate role for herself in *“safeguarding”* the process:

*“I do find it difficult to feel like I have a role in influencing that output* (the guideline)*, I’m obviously not a doctor. I think it would be unrealistic for anyone to expect me to put my hand up and say oh actually I think you should use* (intervention) *because that is not what my role is. But, I think I can be there to see that the way that the committee make their decisions make sense, [...] to, to see that we are playing by the rules if you see what I mean and not so… rather than have that input in terms of the medical side, so I do still have a role but it is different.”* Jennifer, GC9 (interview)

During interviews, several lay members questioned their role in technical discussions. For example, Ben, a lay member from GC6 reflected: “*it would be interesting to know what NICE expect of a lay person going into this very technical, very medical orientated process.*”

However, there was one lay member sub-group who did not appear to struggle with the technical content and scientific language: lay members representing relevant patient charities. Whilst these lay members did sometimes have difficulty with understanding their role and the process of guideline development (described by Grace and Frances earlier), when compared to lay members with lived experience of a condition, members from patient charities did not seem to struggle with the technical content of the guideline development. These *professional* lay members were often able to understand the scientific content and language they were presented with during the guideline development process. Lisa, a lay member from GC4 who worked for a patient charity, and was observed engaging throughout the process, including during the early phase meetings and health outcome selection, explained her position:

“(my perspective is) *very different to other lay members because I have had to develop an unbelievably detailed knowledge of* (disease)*, its treatments, its diagnosis and all the rest of it […] If I didn’t have that I would be lost in that process […] if I was just a member of the public I have no idea how I would necessarily get to grips with all the information that is presented. Or sometimes even understand the discussion.”* Lisa, GC4 (interview)

During observations, Lisa often suggested certain search terms including health outcomes in early meetings such as various adverse events associated with the treatments reviewed in GC4, asked questions or discussed points with other committee members. Nevertheless, like others, Lisa predominantly focused her interview reflections on her involvement in the later phase of clinical guideline development and did not explicitly mention her contributions in earlier meetings about health outcome selection. However, she did distinguish between her knowledge, developed through her work, and that of a ‘member of the public’*.* She therefore had insight into how her professional role enabled her to be involved throughout the clinical guideline development process, in a way that was different from other ‘lay’ members.

#### Assumed differences in priorities

In one particular guideline development, GC9, lay involvement appeared limited by concern on the part of health professionals that the priorities of lay members do not always converge with the best interests of patients. This concern seemed to restrict lay member involvement in health outcome selection.

The GC9 patient population is highly vulnerable and typically needs urgent intensive care; these patients as babies, are unable to represent themselves and thus a *“proxy”,* such as a family member, acts as the patient’s representative. Family members, typically the primary caregiver of the baby, thus made up the lay membership of this committee. During observations of GC9, health professionals talked frequently about their *“moral and ethical obligation”* and *“duty of care”* regarding patients. When developing the clinical guideline, they frequently mentioned this sense of obligation, particularly when discussing the quality of evidence and their own practice. However, whilst health professionals voiced their perspectives and asked each other questions, they rarely asked lay members for their opinions. Cecilia, a health professional on the committee, explained this “*duty of care*” in the context of GC9 as *“proxies often are having to make decisions for (the patient) but the proxies aren’t the actual patients […] sometimes the duty of care is more to the patient, independent of what the proxy might believe.”* As Cecilia’s comment indicates, she saw the interests and needs of patients as sometimes distinct to those of the lay members and therefore had doubts about how relevant the views of lay members were to the guideline.

Observed discussions among health professionals in committee meetings for this guideline indicated that they also believed that the content was too clinically driven for lay members to understand. An example of this occurred when discussing the search terms for an evidence review protocol:

*“Doug (a health professional) was stressing “it has to be clinically important outcomes and outcomes that will not heal with time, lay members won’t know about those, they don’t care.” The other health professionals appeared to concur with this statement as they nodded and murmured their agreement. The lay members continued to sit in silence.”* Fieldnotes from GC9, meeting 4.

Doug here restricted the involvement of lay members before they had chance to express an opinion regarding health outcomes. However, immediately after Doug’s intervention the chair of the committee asked Jennifer, the lay member, for her opinion. Having been silent up to that point, Jennifer suggested a health outcome, albeit in lay terms. One of the health professionals then translated Jennifer’s suggestion into a clinically recognised term that was relevant to the health intervention under discussion. During her interview Jennifer praised the chair of GC9 and the support he provided:

“*I think Andrew is a great chair, [...] he is respectful and he can keep everybody in check. He knows when to bring people in, and he recognises you know when people have something to say….”* Jennifer, GC9 (interview)

During interviews, other lay members praised the committee chairs for their support and guidance. This included recalling instances when chairs ensured the use of plain language in committee meetings, it provided, in those instances, the opportunity for lay members to get involved, and contextualised the clinical guideline content in a patient relevant manner.

### Achieving lay member input

As outlined in three instances described in the sections above (described by experiences of Joan, Lisa and Jennifer), it is possible to achieve lay involvement in health outcome selection. Apart from Lisa, these examples were underpinned by lay members being given the opportunity and support by other committee members and the chairs. In what follows, we present a fourth instance in which lay involvement occurred and indirectly led to relevant health outcome selection.

Richard was a lay member in GC4, with lived experience of the health condition as a patient. By Richard’s own admission he struggled with various aspects of the clinical guideline development process and questioned the influence of his role within the committee. He was mostly silent during meetings and usually only spoke when invited to do so by other committee members. It was following such an invitation that Richard’s input helped the committee to resolve a dilemma regarding what search topics to include in an evidence review protocol:

*The discussion returned to “self-management strategies” and if it should be included in the evidence review protocol. The health professionals who were engaging in this discussion were divided, with some completely for its inclusion and others opposed to it completely. At this point Richard was asked for his opinion by the chair of the committee. He spoke in favour of “self-management strategies” and the positive aspects they carried for patients like himself. After some follow-up questions to Richard from various health professionals and some group discussion it was agreed to include “self-management strategies.* Fieldnotes from G4, meeting 3

Richard’s input provided the impetus to include *“self-management strategies”* as an intervention in the evidence review protocol. Subsequently, the health professionals and technical team members then determined search terms, including health outcomes, in line with the focus on self-management. While Richard did not suggest any health outcomes directly, his perspective resolved a point of conflict between other committee members. By inviting him to share his opinions in a way that was meaningful, the chair signalled that Richard’s perspective was important. In turn, this encouraged other committee members to be receptive to Richard’s *“lay”* opinion.

Richard’s case echoes the dynamics that occurred in two of the instances described earlier, in which lay members were invited to contribute their perspective, experience and opinion by either the chair, technical teams or health professionals and supported by various members of the committee. These inputs were subsequently translated into meaningful search terms for the evidence review protocol.

## Discussion

### Summary of findings

Our findings show that lay involvement in health outcome selection during clinical guideline development was limited due to the guideline development timeframe, technical content of the language used, and concern about the potential differences in priorities between health professionals and lay members who are acting as proxies for the patient. However, findings from this study also indicate that lay involvement is achievable and that continued guidance and support could enhance it, not only in health outcome selection, but also in the overall clinical guideline development process. Further, it is important to note, that this process must be considerate to and accessible to all lay members, regardless of their level of previous experience or professionalism in the healthcare setting.

Our findings reveal a disconnect between how clinical guideline development operates in terms of the technical content, timeframe for production and the hierarchy of roles and tasks and the roles of the lay member. Previous research suggests the technical nature, content and language of clinical guidelines [42, 43], coupled with a lack of training and understanding of scientific methods and guideline development processes [44, 45], can be a barrier to patient and public involvement. These studies point to further training as a potential solution, but caution is needed here. Lay members are invited to committees by virtue of their experiential knowledge [46] and should not be expected to come with scientific knowledge and technical language. However, in its current set-up, when involving lay members, guideline developers are inviting them into the world where research activities and findings can dominate. Thus, we need to ensure that these meetings stay true to their original purpose: to be an intersection in which practice, research and service user worlds can meet, to discuss and develop guidelines. We should also consider support and guidance appropriate to the various roles that inhabit this space. This would help to integrate lay members and enable their meaningful input into clinical guidelines, rather than relying solely on training lay members in scientific processes and language.

Our findings also show that committee members gained confidence to participate in committee discussions more actively overtime. However, this confidence often came too late to influence outcomes. This reflects previous research and theory on how groups and organisations go through different stages of growth and development as they come together and familiarise themselves with each other and their context [47–49]. We therefore argue that procedural changes should be considered. Guideline development via discussion and consensus is ideally an iterative process. However, the procedures and processes currently in use are linear, with set timelines and targets [50]. This linearity seemed to restrict not only lay member involvement, but also health professionals, particularly in the early phase and in health outcome selection. Guideline developers should consider more flexible timelines and methods to support committee members.

Our findings suggest the chair of a committee can serve as a bridge between lay members and health professionals by inviting and facilitating input from the lay perspective. Thus, it is essential that committee chairs have the appropriate skills, support and training. On occasions when we saw lay members become involved in health outcome selection, the other stakeholders acted as translators, articulating the lay input in clinically relevant terms. This highlights the importance of ensuring all stakeholders involved are aware of the importance of lay member input and encouraged to support it. Training all committee members, including health professionals to communicate in plain language, will serve to further include lay members in a meaningful manner. Training of all committee members in the importance of health outcome selection could also further strengthen lay member involvement in this area (Table 3). By drawing on examples in this study where lay members did influence the health outcomes selected, we have identified several processes which help to facilitate and support their involvement, which we summarise as pointers for clinical guideline developers to consider in Table 3. This includes the importance of appropriate and relevant language, engagement and invitation to participate**,** training and support for all committee members and considering the development timeline. It is likely that a combination of these is required to support lay member involvement in health outcome selection and clinical guideline development more generally [7, 42, 45].

**Table 3:** Summary of the pointers and recommendations clinical guideline developers should consider when including lay members in their guideline developments

Pointers for supporting lay involvement in clinical guideline development
**Language**
Clinical guideline developers should ensure the language used in all aspects of the guideline development is accessible to *all* committee members. *Specific suggestions include: ensuring the use of plain language during meetings, avoiding scientific or medical abbreviations, translation of scientific literature and content into plain language formats as needed*
**Engagement and invitation**
Clinical guideline developers should continue to seek ways of engaging with lay members (and other committee members as appropriate) as early in the development process as possible. *This can include: inviting lay members to speak during meetings, directly seeking their opinions during discussion, endeavouring to ensure lay members understand the context and content of the development process*
**Training and support**
It is important that clinical guideline developers continue to understand and embrace the importance of providing training opportunities to the various members of the committee. Training should include the importance of lay member involvement and outcome selection. *This includes training for: the chair of the committee, health professionals, technical team members and lay members themselves. Opportunities for this could include expanding on the outcome section of the PIP lay member training session or communicating it at committee level in the early meetings*
**Timeline**
Clinical guideline developers should explore whether there is flexibility to the development timeline. *Specific suggestions include: seeking input to outcome selection at other junctures*, *establishing “break-out” working group about specific topics, increasing the timeframe for guideline development by having pre-meetings or engagements with committee members*
**Other methods and resources**
Clinical guideline developers could consider seeking alternative and complementary methods of collecting lay member input which can be used to inform committee meetings, specifically in relation to health outcomes. *This could include using other qualitative methods like focus groups and already existing resources such as core outcome sets (COS)*

Other resources could also be considered in addressing the challenges in integrating lay members perspectives in outcome selection. Core outcome sets (COS) are “an agreed standardised set of outcomes that should be measured and reported, as a minimum, in all clinical trials in specific areas of health or health care” [51]. NICE technical team members could search for COS studies [52] that are appropriate to the remit and scope of the guideline being developed [25]. As COS are increasingly developed with input from patients and members of the public [53], their use in the evidence review protocols could help ensure the perspectives of patients are incorporated beyond those of the lay members on the committee. Upstream of evidence review protocols, COS should also be considered at the scoping stage, where stakeholders, including patient representatives can be invited to comment on its applicability to the guideline development, including flagging any missing relevant outcomes. Further lay input could include written patient statements as used in health technology assessments [46] for committees to consider. Qualitative evidence on patients’ perspectives could similarly provide insights for committees to consider [8, 42, 44] and this could be conveyed via a trained patient liaison person or representatives if needed. Such methods could also help overcome the unique challenges of facilitating proxy lay members in clinical guideline development as seen in GC9.

### Strengths and limitations

This study has provided insights into lay members’ influence in clinical guideline development, particularly health outcome selection. Health outcome selection was not a particularly salient topic for interviewees, nor particularly visible during observations. However, by sampling from a range of clinical guidelines we were able to understand why lay involvement may be limited in this respect and identified processes that could facilitate lay member involvement.

This study only describes the experiences of participants who agreed to be interviewed and observations from clinical guidelines and other meetings that we could access. Thus, while saturation was reached within our sample, we note that the experiences and perspectives gathered in this study may not be typical of guideline development either within NICE or more broadly. However, as we sampled a range of clinical guideline meetings and lay members, we anticipate that our findings will be broadly transferable to other clinical guideline development programmes. Furthermore, while this study explored lay member involvement, our findings will also benefit the involvement of other committee members such as health professionals.

This study explored PPI at NICE, within the context of their frameworks and processes to facilitate inclusion of lay members. We acknowledge that this may be different to how PPI is characterised more broadly within health research, but it is beyond the scope and remit of this paper to comment on such differences. However, by exploring PPI within the remit of NICE, we have provided a detailed account of what unfolded within the particular guideline development that we observed. We anticipate the findings will be of use more broadly to both NICE and other clinical guideline development programmes.

This study captured and characterised the experiences and perspectives of lay members as defined and recruited for by NICE, which encompasses both professional and non-professional lay members, who had varying levels of previous experience within the healthcare setting. As a result, our findings need to be understood within that remit. However, by sampling widely across a range of guidelines we sought to ensure that a broad range of perspectives are represented within this study and its findings.

A limitation often associated with ethnography is the challenge of separating the ethnographic subject and the researcher's analysis [54]. However, by taking a pragmatic approach and utilising an interpretivist lens, we are acknowledging that the researcher and their study population are interdependent and mutually interactive [37] and our analysis and reflexivity is grounded in this concept.

## Conclusion

Challenges to lay member input during the outcome selection phase of clinical guideline development exist. Yet, this study has also found that such input is possible and has identified how it can be achieved. The findings will support future guideline developers working towards enhanced meaningful lay involvement in guideline development and health outcome selection. Future research should consider exploring what is means to be a lay member and the different types of lay member that exists in these settings in terms of professionalism and previous health research setting experience. Future research should also investigate potential differences between what is meant by PPI in health research more broadly versus how lay member inclusion is implemented at NICE, and what lessons and learnings, if any, are transferable between them.

## Supplementary Information

**Additional file 1.**

## Data Availability

The datasets generated and analysed during the current study are not publicly available due to the sensitive and personal nature of the data collected and as participants only consented to their data being used in relevant future studies, but are available from the corresponding author on reasonable request.

## Abbreviations

COS: Core outcome sets
NICE: National Institute of Health and Care Excellence
NHS: National Health Service
PICO: Population- Intervention-Comparator- Outcome
PIP: Public Involvement Programme
PPI: Patient and Public Involvement
