# Supplementary material for: Enhancing patient and public contribution in health outcome selection during clinical guideline development: an ethnographic study
Source: BMC Health Serv Res. 2022 Mar 18;22:361. doi: 10.1186/s12913-022-07736-6 (PMC8933885; doi:10.1186/s12913-022-07736-6)
Supplement: Supplementary file 1 — Additional file 1. [file 12913_2022_7736_MOESM1_ESM.docx]

Appendix

1. Semi-structured interview guide for committee members involved in clinical guideline development.

**Topic guide (chronological)**

1. **Q.** Could you talk me through how you became involved in the clinical guideline development?

**Prompts**: *How did you become aware of the study? –recruitment advert, methods*

1. **Q.** Can you tell me about how you prepared for the guideline development meetings? (both before and throughout the meetings)

**Prompts:** *Can you tell me about any support provided for the first meeting?*

*What was included in this?*

**For lay-members:** What were your experiences of the lay member training sessions

1. **Q.** Can you tell me about your experiences of being involved in the guideline development meetings?

**Prompts:** *Thinking back to the very start, what did you expect being involved in this guideline development would be like?*

*How has the reality compared with your expectation?*

*How do you think your involvement affected the guideline development? (If so, in what way? (Can you tell me a little bit more about that?))*

*How was your relationship with the rest of the committee? (Were there any challenges? If so, how were these challenges* *resolved?)*

*Is there anything that stands out about the meetings?*

*What’s your understanding of what was expected of you in relation to your participation in the guideline development meetings?*

***For lay members:*** *How did you experience having other lay members on the committee with you?*

*What was it like being a part of a committee with health professionals?*

**For healthcare professionals:** *What was your experience of having lay members on the committee with you?*

1. **Final Questions:**

**Q.** Do you have any suggestions for improving the process?

**Q.** Is there something else that is important to you that we haven’t talked about today?
